# Prognostic significance of systemic immune-inflammation index in patients with ovarian cancer: a meta-analysis

**DOI:** 10.3389/fonc.2023.1193962

**Published:** 2023-06-05

**Authors:** Huaying Mao, Fan Yang

**Affiliations:** ^1^ Clinical Laboratory, Huzhou Central Hospital, Affiliated Central Hospital of Huzhou University, Huzhou, Zhejiang, China; ^2^ Clinical Laboratory, Huzhou Maternity and Child Health Care Hospital, Huzhou, Zhejiang, China

**Keywords:** SII, ovarian cancer, meta-analysis, prognosis, survival

## Abstract

**Background:**

The prognosis of several malignancies has been influenced by the systemic immune-inflammation index (SII); however, its association with the prognostic outcome of ovarian cancer (OC) remains controversial. The present meta-analysis focused on the systemic and comprehensive identification of the role of SII in predicting OC prognosis.

**Methods:**

We searched the Web of Science, PubMed, Cochrane Library, Embase, and China National Knowledge Infrastructure (CNKI) from inception until March 6, 2023. To predict the prognostic value of SII for overall survival (OS) and progression-free survival (PFS) in patients with OC, we calculated pooled hazard ratios (HRs) and corresponding 95% confidence intervals (CIs).

**Results:**

The meta-analysis included six studies involving 1546 patients. The combined results showed that a high SII was significantly associated with poor OS (HR=2.70, 95% CI=1.98–3.67, p<0.001) and poor PFS (HR=2.71, 95% CI=1.78–4.12, p<0.001) in OC patients. These results were confirmed using subgroup and sensitivity analyses.

**Conclusion:**

Our results concluded that a high SII significantly predicted poor OS and PFS in patients with OC. Therefore, it can be speculated that the SII may have an independent effect on the prognosis of OC.

## Introduction

There are several histopathological subtypes of ovarian neoplasms, with epithelial ovarian cancer (OC) accounting for the majority (about 90%) of them (1). Globally, OC is ranked 7th and 8th in terms of cancer morbidity and mortality in women, respectively (2). According to GLOBOCAN estimates, there were 313 959 newly diagnosed cases of OC and 207 252 deaths due to OC in 2020 worldwide (3). Approximately 60% of patients with OC are at an advanced stage or have widespread intra-abdominal metastatic disease at the time of initial diagnosis because there are no specific early symptoms (4). The treatments for OC include surgical resection, radiotherapy, chemotherapy, immunotherapy, and targeted therapy (5, 6). Although systemic treatment of OC is becoming increasingly advanced, there has been no significant improvement in the prognosis of OC patients. The five-year survival rate of patients with early-stage OC is 95%, whereas it is less than 30% in patients with advanced OC (stage III or IV) (7). The poor prognosis of OC is partially due to a lack of effective prognostic markers (8). Consequently, it is important to identify new and accurate biomarkers for predicting OC.

Recently, inflammation was found to exert a critical effect on tumor development, recurrence, and metastasis (9). Existing studies have found that systemic inflammation-related indicators can adequately evaluate and predict tumor development and prognosis. These inflammatory response markers include the neutrophil-to-lymphocyte ratio (NLR) (10), platelet-to-lymphocyte ratio (PLR) (11), lymphocyte-to-monocyte ratio (LMR) (12), and the modified Glasgow prognostic score (mGPS) (13). The systemic immune inflammation index (SII) is a blood test-based inflammatory marker that is determined as follows: SII = neutrophil count × platelet count/lymphocyte count. The SII is valuable for predicting cancer prognosis, including colorectal cancer (CRC) (14), esophageal cancer (15), nasopharyngeal carcinoma (NPC) (16), bladder cancer (17), gastric cancer (GC) (18), and neuroblastoma (19). For example, an elevated SII index could predict the diagnosis of postoperative infectious complications and the long-term prognosis of patients with CRC (14). Preoperative SII could predict the survival of patients with thymoma who underwent radical resection (20). A recent study showed that in patients with NPC, SII is a promising indicator for predicting survival, especially the risk of uncontrolled recurrence (16). Some existing studies have analyzed the role of SII in predicting OC prognosis. However, their results remain conflicting (21–26). Few studies have identified high SII as a significant prognostic factor for survival in OC (21, 25, 26). In contrast, other studies have found an insignificant relationship between the SII and prognostic outcomes of OC (22). Consequently, we conducted a comprehensive literature review to identify the precise role of SII in the prognosis of patients with OC.

## Materials and methods

### Study guideline

This work was conducted and reported in line with the guidelines from the Preferred Reporting Items for Systematic Reviews and Meta-Analyses (PRISMA) (27).

### Search strategy

This study comprehensively and thoroughly searched PubMed, Embase, Web of Science, the China National Knowledge Infrastructure (CNKI), and Cochrane Library databases from inception until May 10, 2023. T The search terms were as follows: (systemic immune-inflammation index or systemic immune-inflammatory index or SII), and (ovarian cancer or ovarian carcinoma or carcinoma of the ovary or ovarian neoplasm or ovary cancer or ovary tumor). There were no restrictions on publication language. Reference lists of the collected articles were manually searched for additional candidate articles.

### Selection criteria

We developed the selection criteria based on previous literature (28–30). Studies that met the following criteria were included in the analysis (1): the diagnosis of OC was histologically or pathologically confirmed; (2) studies reporting the hazard ratios (HRs) together with corresponding 95% confidence intervals (CIs) concerning SII before treatment, as well as survival outcomes in OC; (3) the threshold SII was identified; and (4) studies published in Chinese or English. The exclusion criteria were: (1) reviews, case reports, letters, meeting abstracts, or comments; (2) studies with overlapping patients; and (3) animal studies.

### Data collection

Eligible studies were selected, and the relevant information was collected independently by two researchers (HM and FY). Any differences between the authors were resolved through mutual negotiation until a consensus was reached. The following information was collected: first author, publication year, country, sample size, study period, age, FIGO stage, treatment, survival outcomes, threshold SII, study center, follow-up, and HRs with 95% CIs.

### Quality evaluation

Methodological quality was scored using the Newcastle-Ottawa Scale (NOS) by two independent investigators (HM and FY) (31). The NOS covers three domains: selection quality (0–4 points), comparability (0–2 points), outcome evaluation, and adequacy of follow-up (0–3 points), with a total score ranging between 0 and 9. The scores > 6 indicated high-quality studies.

### Statistical analysis

We computed pooled HRs and corresponding 95% CIs to estimate the significance of SII in predicting overall survival (OS) progression-free survival (PFS) in patients with OC. Cochran’s Q test (32) and I^2^ test (33) were used to assess statistical heterogeneity across studies. As I^2^ > 50% and p < 0.10 represent substantial heterogeneity, the random-effects model (REM) was applied. Otherwise, the fixed-effects model (FEM) was adopted. Sources of heterogeneity were detected using subgroup analysis. Furthermore, each study was eliminated in sequence during the sensitivity analysis to detect any influence on the pooled results. Publication bias was quantified using Begg’s and Egger’s tests. Statistical significance was set at p < 0.05. All statistical analyses were performed using Stats software (version 12.0; StataCorp LP, College Station, TX, USA).

### Ethnics statement

This investigation was based on data from previously published studies. Therefore, ethical approval was not required.

## Results

### Process of literature selection

**Figure 1:**
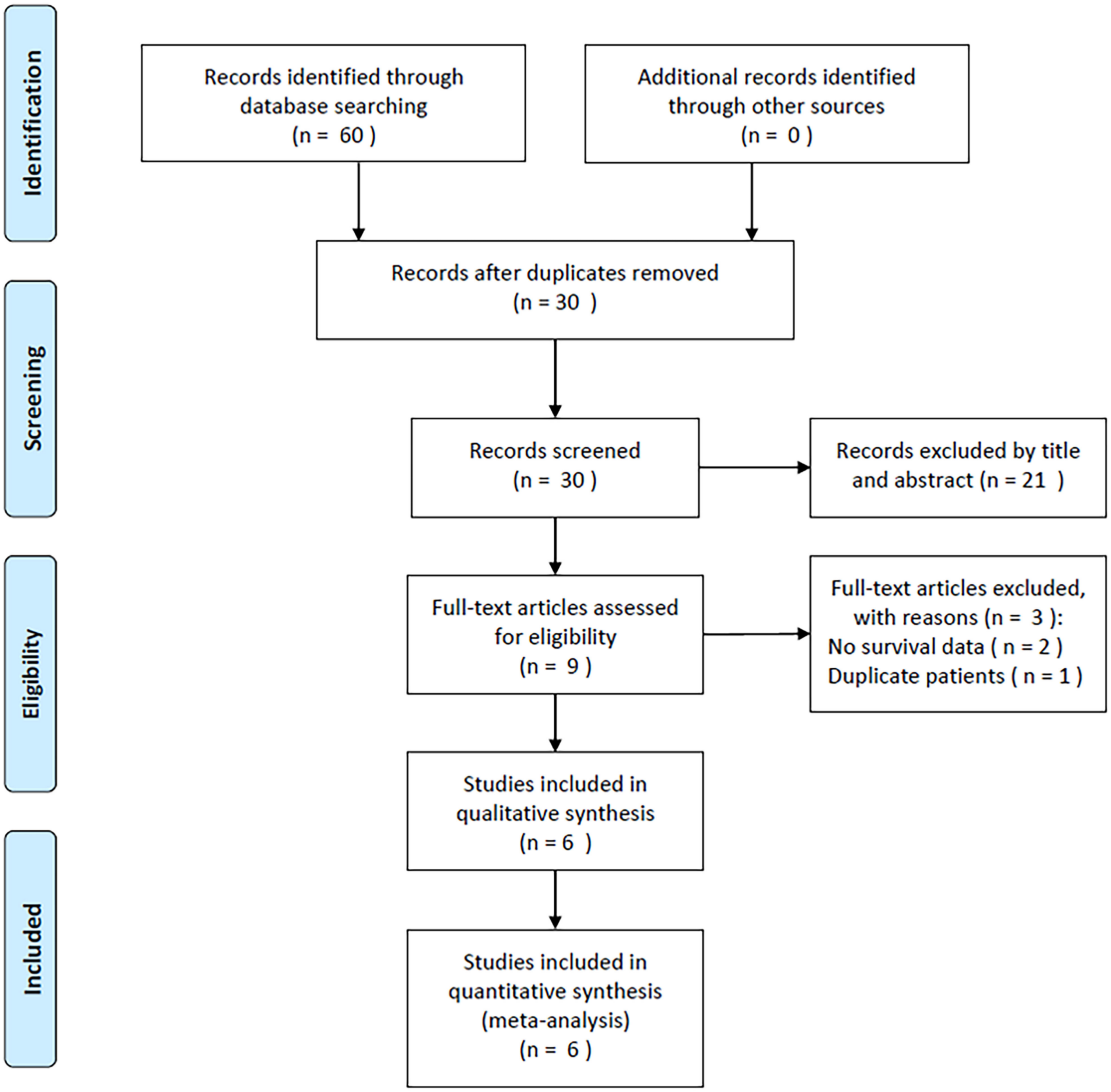
PRISMA flow diagram outlining the literature search process.

In total, 60 studies were obtained through a preliminary search, and duplicates were removed to obtain 30 studies (**Figure 1**). After title and abstract reviews, 21 irrelevant studies were excluded. Nine studies were assessed by reading the full texts. Three studies were subsequently eliminated owing to the unavailability of survival data (n=2) and overlapping patient recruitment (n=1). Finally, the meta-analysis included six studies involving 1546 patients (21–26) (**Figure 1**).

### Study characteristics

**Table 1** summarizes the basic features of the selected studies. The publication years of the enrolled articles, which had a retrospective design, were between 2019 and 2023. Five articles were published in English (21, 22, 24–26) and one in Chinese (23). Three studies were conducted in China (21, 23, 25), two in Italy (22, 26), and one in India (24). The sample sizes were 49–553 (median, 233.5). Three studies included patients with OC who underwent chemotherapy (22–24), two studies included patients treated surgically (21, 26), and one study included patients treated using surgery and chemotherapy (25). The SII threshold was 612–1000 (median: 715.5). Five studies, which included 1438 patients, reported a relationship between SII and OS (21, 22, 24–26). All six studies involving 1546 patients confirmed the role of SII in PFS prediction (21–26). Five studies were performed at a single center (21, 23–26) and one was a multicenter study (22). Moreover, the NOS scores were 6–9, indicating their high quality (**Table 1**).

**Table 1:** Characteristics of included studies in this meta-analysis.

Study	Year	Country	Sample size	Study period	Age (year) Median (range)	FIGO stage	Treatment	Cut-off value	Survival endpoints	Study center	Follow-up (month) Median (range)	NOS score
Nie, D.	2019	China	553	2009-2012	53 (22-83)	I-IV	Surgery	612	OS, PFS	Single center	46 (3-95)	8
Farolfi, A.	2020	Italy	375	2007-2015	58 (31-83)	III-IV	Chemotherapy	730	OS, PFS	Multicenter	1-125	9
Liu, J.	2020	China	108	2017-2019	57	II-IV	Chemotherapy	701	PFS	Single center	1-12	7
Goenka, L.	2022	India	49	2015-2019	NA	III-IV	Chemotherapy	639	OS, PFS	Single center	7	6
Wang, J.	2022	China	102	2017-2019	56	III	Surgery+ chemotherapy	872	OS, PFS	Single center	1-36	8
Bizzarri, N.	2023	Italy	359	2012-2019	54 (21-93)	I-III	Surgery	1000	OS, PFS	Single center	31	7

OS, overall survival; PFS, progression-free survival; FIGO, International Federation of Gynecology and Obstetrics; NOS, Newcastle-Ottawa Scale; NA, not available.

### SII and OS

**Figure 2:**
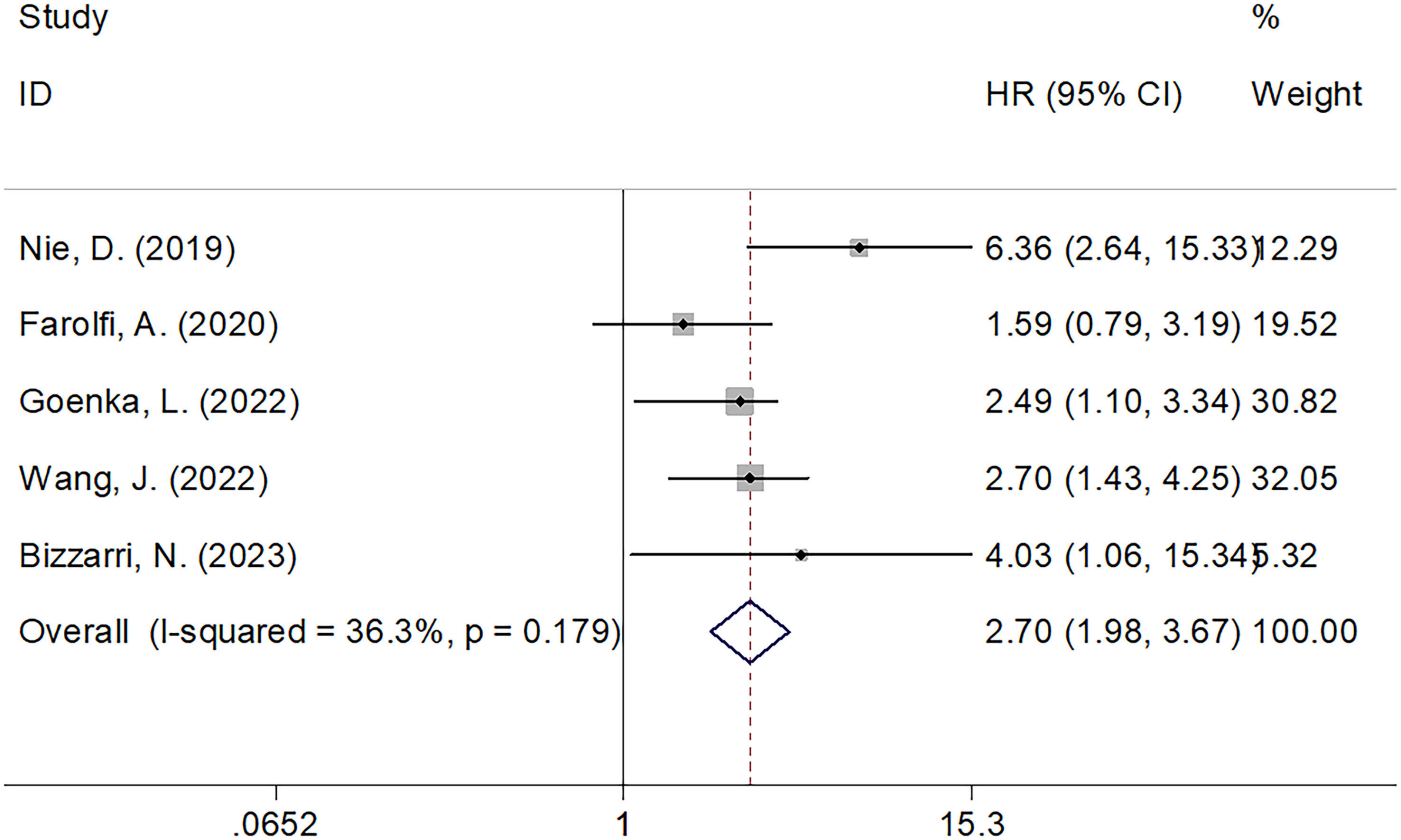
Forest plots evaluating the HRs of SII in patients with ovarian cancer for OS.

Five studies comprising 1438 patients (21, 22, 24–26) mentioned SII’s performance in predicting the OS of patients with OC. The FEM was used because of the non-obvious heterogeneity (I^2 =^ 36.3%, p=0.179). According to **Figure 2**; **Table 2**, pooled HR=2.70, 95% CI=1.98–3.67, p<0.001 were obtained. These suggested that an increased SII remarkably predicted a poor OS. Subgroup analyses were performed based on country, sample size, treatment, study center, and threshold values. As shown in **Table 2**, the role of SII in predicting OS remained unchanged by country, sample size, treatment, or study center (p<0.05). Moreover, elevated SII still significantly predicted OS among single-center studies (HR=3.06, 95% CI=2.17–4.32, p<0.001; **Table 2**).

**Table 2:** The subgroup analysis of prognostic role of SII for OS in OC.

Subgroups	No. of studies	No. of patients	Effects model	HR (95%CI)	p	Heterogeneity I^2^ (%) Ph	
Total	5	1,438	FEM	2.70 (1.98-3.67)	<0.001	36.3	0.179
Country							
China	2	655	REM	3.85 (1.68-8.82)	0.001	62.0	0.105
Italy	2	734	FEM	1.94 (1.05-3.60)	0.036	31.6	0.227
India	1	49	–	2.49 (1.43-4.34)	0.001	–	–
Sample size							
<200	2	151	FEM	2.59 (1.76-3.83)	<0.001	0	0.838
≥200	3	1,287	REM	3.27 (1.27-8.46)	0.014	67.4	0.046
Treatment							
Chemotherapy	2	424	FEM	2.09 (1.36-3.23)	0.001	0	0.324
Surgery	2	912	FEM	5.54 (2.66-11.55)	<0.001	0	0.576
Surgery + chemotherapy	1	102	–	2.70 (1.57-4.65)	<0.001	–	–
Study center							
Single center	4	1,063	FEM	3.06 (2.17-4.32)	<0.001	15.6	0.314
Multicenter	1	375	–	1.59 (0.79-3.20)	0.193	–	–
Cut-off value							
<720	2	602	REM	3.73 (1.50-9.27)	0.005	68.0	0.077
≥720	3	836	FEM	2.34 (1.55-3.52)	<0.001	3.8	0.354

OS, overall survival; SII, systemic immune-inflammation index; OC, ovarian cancer; FEM, fixed-effects model; REM, random-effects model.

"-" means blank.

### SII and PFS

**Figure 3:**
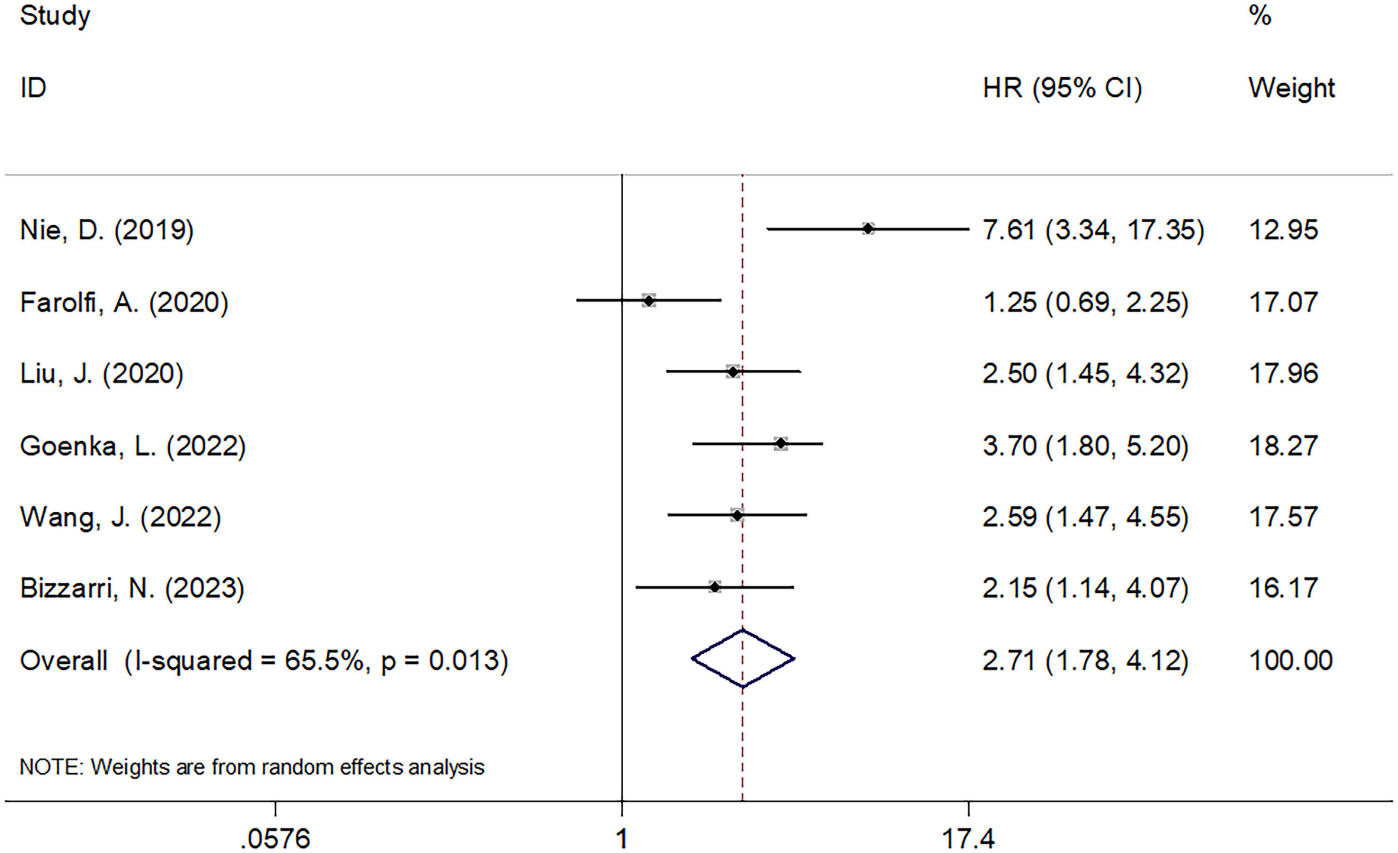
Forest plots evaluating the HRs of SII in patients with ovarian cancer for PFS.

The relationship between the SII and PFS was investigated in all six studies involving 1546 patients (21–26). We used REM because of its significant heterogeneity (I^2 =^ 65.5%, p=0.013). Pooled HR=2.71, 95% CI=1.78–4.12, p<0.001 were obtained, suggesting the obvious relation between increased pretreatment SII and poor PFS of OC (**Table 3**; **Figure 3**). As suggested by the subgroup analysis, an increased SII remarkably predicted poor PFS, regardless of the country, sample size, or threshold (p<0.05; **Table 3**). Furthermore, as shown in **Table 3**, an elevated SII still predicted shortened PFS in studies with a sample size <200 (p<0.001) and in single-center studies (p<0.001).

**Table 3:** The subgroup analysis of prognostic role of SII for PFS in OC.

Subgroups	No. of studies	No. of patients	Effects model	HR (95%CI)	p	Heterogeneity I^2^ (%) Ph	
Total	6	1,546	REM	2.71 (1.78-4.12)	<0.001	65.5	0.013
Country							
China	3	763	REM	3.41 (1.85-6.27)	<0.001	64.0	0.062
Italy	2	734	FEM	1.61 (1.04-2.48)	0.032	33.0	0.022
India	1	49	–	3.70 (2.18-6.29)	<0.001	–	–
Sample size							
<200	3	259	FEM	2.90 (2.12-3.98)	<0.001	0	0.535
≥200	3	1,287	REM	2.63 (1.00-6.93)	0.050	83.6	0.002
Treatment							
Chemotherapy	3	532	REM	2.29 (1.24-4.21)	0.008	72.4	0.027
Surgery	2	912	REM	3.93 (1.14-13.56)	0.030	82.3	0.017
Surgery + chemotherapy	1	102	–	2.59 (1.47-4.55)	0.001	–	–
Study center							
Single center	5	1,171	FEM	3.05 (2.33-3.98)	<0.001	44.7	0.124
Multicenter	1	375	–	1.25 (0.69-2.26)	0.459	–	–
Cut-off value							
<720	3	710	REM	3.85 (2.20-6.73)	<0.001	59.1	0.087
≥720	3	836	FEM	1.91 (1.36-2.70)	<0.001	37.6	0.201

PFS, progression-free survival; SII, systemic immune-inflammation index; OC, ovarian cancer; FEM, fixed-effects model; REM, random-effects model.

"-" means blank.

### Sensitivity analysis

**Figure 4:**
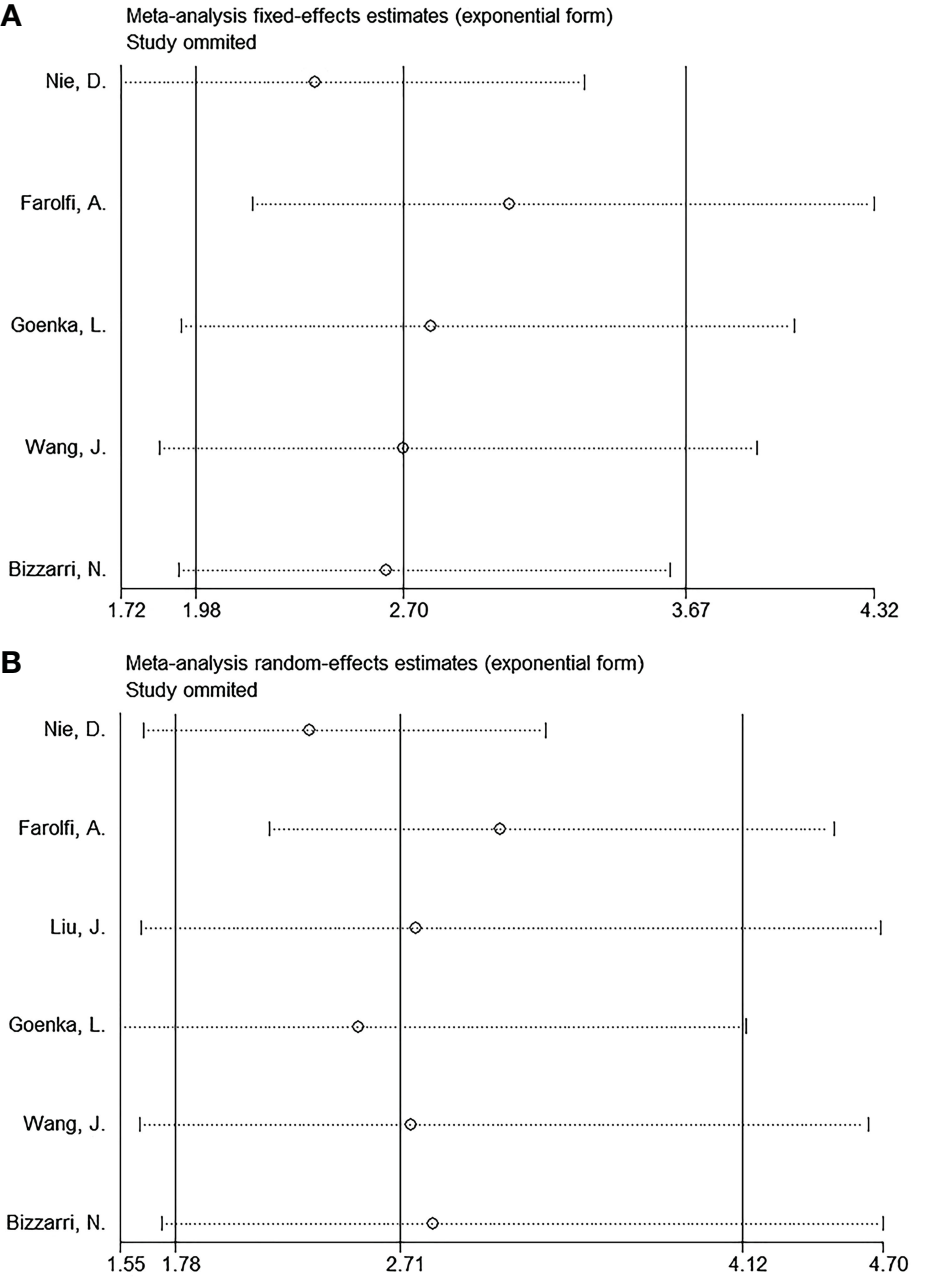
Sensitivity analysis. **(A)** OS, and **(B)** PFS.

We determined whether an individual study affected the overall results by performing a sensitivity analysis. None of the articles significantly affected the magnitude of the combined effects on OS and PFS after deletion, indicating that the results were reliable (**Figure 4**).

### Publication bias

**Figure 5:**
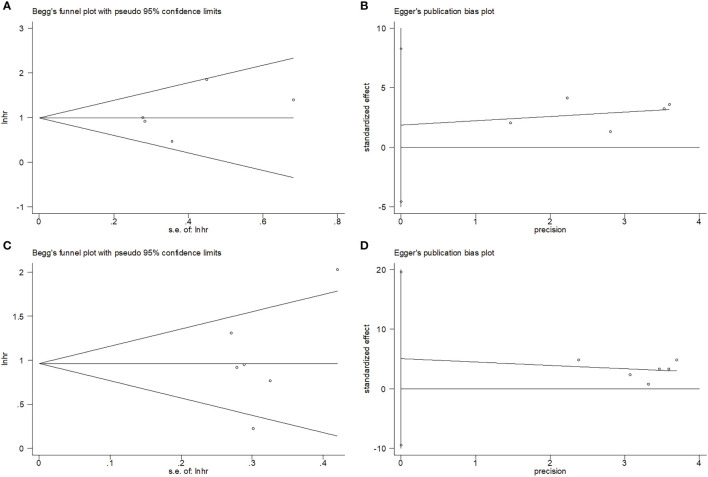
Publication test by Begg’ test and Egger’s test. **(A)** Begg’s test for OS, p=0.806; **(B)** Egger’s test for OS, p=0.424; **(C)** Begg’s test for PFS, p=0.851; and **(D)** Egger’s test for PFS, p=0.388.

Begg’s test and Egger’s test were used to test publication bias. The funnel plots in **Figure 5** show that OS and PFS were symmetric. The OS and PFS obtained using Begg’s and Egger’s tests were p=0.806 and 0.424, and p=0.851 and 0.388, respectively. These suggested the pooled outcomes were due to the absence of publication bias (**Figure 5**).

## Discussion

The role of SII in predicting OC prognosis remains controversial in existing investigations (21–26). This study collected information from six studies involving 1546 patients and quantitatively identified the role of SII in predicting OS and PFS in patients with OC. Based on the pooled data, an increased SII markedly predicted OS and PFS. Additionally, the SII achieved creditable performance in predicting prognosis, as suggested by sensitivity analysis and publication bias. The SII is a blood-test-derived biomarker and is cheap and simple because neutrophil, lymphocyte, and platelet counts are routinely examined in clinical settings. Therefore, the SII may be an effective inflammatory index to estimate the short- and long-term survival of patients with OC owing to its good discriminatory value. To our knowledge, the present work is the first meta-analysis to investigate the role of SII in predicting OC prognosis.

There is growing evidence that immune cells play an essential role in inflammation, contributing to the generation of chemokines and cytokines that promote tumorigenesis, development, metastasis, and angiogenesis (34, 35). Simultaneously, SII is an inflammatory index derived from neutrophil, lymphocyte, and platelet counts. Therefore, a higher SII may result from higher neutrophil, platelet, and/or lower lymphocyte counts. Moreover, the significant connection between SII and the poor prognosis of OC is interpreted below. First, neutrophils are the first line of natural immune defense that is mobilized by the body when an infection occurs. Previous studies have established that neutrophils regulate tumor cell growth and metastasis by generating different inflammatory factors, such as OSM, TGF-β, HGF, and CXCL8 (36). Neutrophils can help construct an immune-privileged tumor microenvironment, which facilitates cancer cells to escape immune surveillance (37). Second, adenine and adenylate are released by platelets to protect circulatory cancer cells, inducing epithelial-mesenchymal transformation (EMT) while favoring tumor cell colonization and invasion (38). In addition, platelets may promote distal metastasis by activating the NF-κB and Smad pathways, which protect tumor cells against lysis induced by natural killer cells (39, 40). Third, lymphocytes have an important effect on cellular immunity, tumor immune editing, and immune surveillance. An increase in lymphocytes indicates immune pathway activation (41). Tumor-infiltrating lymphocytes (TILs) enhance the suppression of cancer cell growth, invasion, and cytotoxic cell death, thereby having an important effect on cancer defense (42). Impaired innate immunity may result from lymphocytopenia and suppression of lymphocyte activity induced by the systemic immune response (43). As a result, patients with a higher SII had higher neutrophil/platelet counts and lower lymphocyte counts, which are factors that predict a poor prognosis.

Notably, the subgroup analysis demonstrated that the prognostic role of the SII for OS and PFS was not influenced by the treatment methods (**Tables 2**, **3**). These results have important implications for clinical practice. Treatments for OC include systemic therapies such as surgery, chemotherapy, radiotherapy, targeted therapy, poly (ADP-ribose) polymerase (PARP) inhibitors, and immunotherapy (44). Patients with OC may receive one or more treatments at different disease stages (6). Therefore, SII may be an effective prognostic marker for patients with OC undergoing various therapeutic strategies. The disease stages in the included studies were also not uniform (21–26). One study examined early-stage OC (25), whereas the other four studies examined recurrent/relapsed OC (21–24). We combined these studies for the following reasons: first, all eligible studies were selected based on the same inclusion and exclusion criteria; therefore, they were comparable. Second, the subgroup analysis demonstrated that the prognostic value of the SII was not affected by the various treatment methods.

The SII was calculated as SII = neutrophil count × platelet count/lymphocyte count. Therefore, SII can be considered a combination of NLR and PLR. Moreover, SII is more sensitive than NLR or PLR because it uses the product of neutrophil and platelet count as the numerator. SII is also cost-effective and easily available as other blood test-related markers such as NLR, PLR, LMR, and mGPS. We noticed that the HRs and 95% CIs of Nie’s study (21) were higher than those of the other included studies (22–26). These observations could be attributed to several factors. First, the cut-off value in Nie’s study was the lowest among the included studies (**Table 1**). Second, the patients in Nie’s study underwent surgery, and their survival may have been longer than that of patients undergoing chemotherapy. Therefore, the HRs and 95% CIs in Nie’s study could be higher than those in other studies.

Recently, numerous meta-analyses have analyzed SII’s performance in predicting various cancer types (28, 30, 45–48). In a meta-analysis of 1768 patients, Wang et al. reported that in breast cancer cases (BC), poor OS and disease-free survival (DFS) were considerably correlated with increased SII (48). As discovered by Fu et al. in their meta-analysis of 6925 patients, an increased SII remarkably predicted poor OS and worse DFS in GC (49). According to a recent meta-analysis of 2132 patients, a higher pretreatment SII score predicted poor OS and low cancer-specific survival (CSS), DFS, and PFS in pancreatic cancer (28). In the meta-analysis involving eight studies carried out by Zhou et al., the higher SII remarkably predicted poor OS and extensive-stage among patients with small cell lung cancer (SCLC) (30). As shown by Cao et al. in a meta-analysis of 11 studies, an increased SII before treatment remarkably predicted dismal OS, CSS, PFS, and recurrence-free survival (RFS) inpatients with bladder cancer (50).

However, this study had some limitations. First, there were variations in the treatment of patients with OC in the enrolled studies, possibly affecting survival and causing heterogeneity. Different chemotherapy treatments were administered to 532 patients (22–25), of whom 108 patients received bevacizumab (23), and 423 patients received platinum-based drugs (22, 24, 25). More studies are needed to investigate the prognostic role of SII in patients with OC who received bevacizumab. Neoadjuvant chemotherapy (NACT) is commonly used to treat OC. The prognostic value of SII in patients with OC treated with NACT + surgery needs to be verified. Second, although the SII was markedly related to OS and PFS, the studies had small sample sizes and few publications. Third, retrospective articles were included in the meta-analysis, which may have induced a selection bias. Therefore, large prospective multiregional studies should be conducted to validate our findings.

## Conclusions

In conclusion, the present meta-analysis showed that an increased SII significantly predicted worse OS and PFS in patients with OC. Consequently, we can speculate that SII may have an independent effect on the prognosis of OC.

## Data availability statement

The original contributions presented in the study are included in the article/supplementary material. Further inquiries can be directed to the corresponding author. The data that support the findings of this study are available from the corresponding author upon reasonable request.

## Author contributions

HM and FY contributed to the study design and data collection. Statistical analyses and interpretation of results were performed by HM and FY. HM drafted the manuscript. FY revised the manuscript and edited the language. All authors made the critical revision of the manuscript for important intellectual content. All authors contributed to the article and approved the submitted version.
